# Supplementary material for: Analysis of in vivo infection dynamics using bioluminescent Giardia duodenalis
Source: Infect Immun. 2026 Jun 15;94(7):e00144-26. doi: 10.1128/iai.00144-26 (PMC13367060; doi:10.1128/iai.00144-26)
Supplement: Fig S1 — Map of plasmid. [file iai.00144-26-s0001.pdf]

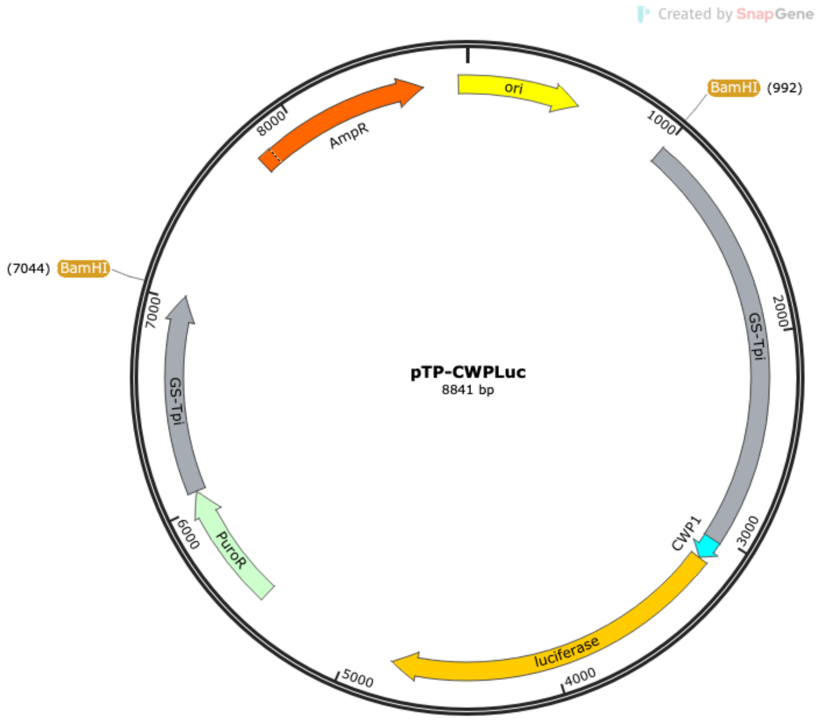

Supplemental Figure 1. Map of pTP-CWPLuc used to integrate the firefly luciferase gene into the TPI locus of *Giardia* isolate GS.
